# The Phenology of Ticks and the Effects of Long-Term Prescribed Burning on Tick Population Dynamics in Southwestern Georgia and Northwestern Florida

**DOI:** 10.1371/journal.pone.0112174

**Published:** 2014-11-06

**Authors:** Elizabeth R. Gleim, L. Mike Conner, Roy D. Berghaus, Michael L. Levin, Galina E. Zemtsova, Michael J. Yabsley

**Affiliations:** 1 Warnell School of Forestry and Natural Resources, University of Georgia, Athens, Georgia, United States of America; 2 Southeastern Cooperative Wildlife Disease Study, College of Veterinary Medicine, University of Georgia, Athens, Georgia, United States of America; 3 Wildlife Laboratory, Joseph W. Jones Ecological Research Center at Ichauway, Newton, Georgia, United States of America; 4 Department of Population Health, College of Veterinary Medicine, University of Georgia, Athens, Georgia, United States of America; 5 Centers for Disease Control and Prevention, Rickettsial Zoonoses Branch, Atlanta, Georgia, United States of America; Washington State University, United States of America

## Abstract

Some tick populations have increased dramatically in the past several decades leading to an increase in the incidence and emergence of tick-borne diseases. Management strategies that can effectively reduce tick populations while better understanding regional tick phenology is needed. One promising management strategy is prescribed burning. However, the efficacy of prescribed burning as a mechanism for tick control is unclear because past studies have provided conflicting data, likely due to a failure of some studies to simulate operational management scenarios and/or account for other predictors of tick abundance. Therefore, our study was conducted to increase knowledge of tick population dynamics relative to long-term prescribed fire management. Furthermore, we targeted a region, southwestern Georgia and northwestern Florida (USA), in which little is known regarding tick dynamics so that basic phenology could be determined. Twenty-one plots with varying burn regimes (burned surrounded by burned [BB], burned surrounded by unburned [BUB], unburned surrounded by burned [UBB], and unburned surrounded by unburned [UBUB]) were sampled monthly for two years while simultaneously collecting data on variables that can affect tick abundance (e.g., host abundance, vegetation structure, and micro- and macro-climatic conditions). In total, 47,185 ticks were collected, of which, 99% were *Amblyomma americanum*, 0.7% were *Ixodes scapularis,* and fewer numbers of *Amblyomma maculatum*, *Ixodes brunneus*, and *Dermacentor variabilis.* Monthly seasonality trends were similar between 2010 and 2011. Long-term prescribed burning consistently and significantly reduced tick counts (overall and specifically for *A. americanum* and *I. scapularis*) regardless of the burn regimes and variables evaluated. Tick species composition varied according to burn regime with *A. americanum* dominating at UBUB, *A. maculatum* at BB, *I. scapularis* at UBB, and a more even composition at BUB. These data indicate that regular prescribed burning is an effective tool for reducing tick populations and ultimately may reduce risk of tick-borne disease.

## Introduction

In the past several decades, numerous novel tick-borne diseases have emerged and the incidence of other tick-borne diseases has increased [1]–[8]. While this increase is likely due to a number of factors, such as increases in reporting, diagnosis and host abundance, one of the primary drivers is thought to be human land modification and management practices [3], [8], [9]. This hypothesis has underscored the importance of understanding how human land management practices affect tick population dynamics as well as identifying methods to control tick populations and reduce human disease risk.

Perhaps one of the most promising methods for controlling tick populations is prescribed burning because it can be applied on a landscape level and is relatively time and cost efficient. Additionally, prescribed burning is a well-accepted form of ecosystem management and wildfire prevention [10]–[12]. In the southeastern United States, the value of prescribed burning is particularly apparent where forests are dominated by fire-adapted or fire–dependent tree species, such as longleaf pines (*Pinus palustris*). Specifically, burns reduce fuel load and cause regeneration and diversification of the understory of forests [11], [13]–[16]. In addition, the immediate regeneration of understory vegetation after a burn and the long-term density and diversity of understory vegetation within regularly burned habitat provides resources for many wildlife species including the endangered or threatened gopher tortoise (*Gopherus polyphemus*), Eastern indigo snake (*Drymarchon couperi*), and red-cockaded woodpecker (*Picoides borealis*) [11], [15], [17]–[18].

Several past studies have been conducted on the impacts of prescribed burning on tick populations. Although there have been conflicting results, the majority of studies concluded that tick populations are reduced immediately after a burn event, but recover to pre-burn abundance within one year [19]–[27]. However, other studies have failed to observe a significant reduction in ticks and some even observed an increase [28]–[30]. These studies often did not simulate real-world management conditions in that burns in many studies were performed only once, over a small area of land, and/or were performed in areas that were previously unburned [19]–[21], [23], [24], [26], [31], [32]. This is in direct contrast to the typical regimen for applying prescribed burns, which are conducted over hundreds to thousands of acres on a regular basis. These discrepancies have clear implications for tick re-establishment after a burn and should be accounted for in an analysis on the impacts of burning on tick populations. Furthermore, previous studies typically did not investigate other factors known to affect tick populations such as host abundance, microclimate and vegetation structure [21], [24]. Failure to account for such factors may explain some of the conflicting results of past studies.

To address impacts of long-term prescribed burning on tick population dynamics and evaluate how prescribed burns might interact with other factors to affect tick abundance, we determined tick abundance, species composition, and seasonality at multiple plots with variable burn histories with >10 years of operational burn management. Additionally, other factors known to potentially affect tick abundance (e.g., host abundance, vegetation structure and micro- and macroclimate) were evaluated at each plot. Collectively, these data provided insight in to the efficacy of long-term prescribed burning for tick control and also revealed phenologies for numerous tick species in southwestern Georgia and northwestern Florida (USA), an area for which there is currently little to no data on ticks or tick-borne pathogens.

## Methods

### Study Area

This study was conducted in southwestern Georgia and northwestern Florida, which is dominated by pine and mixed pine forests and agriculture. Prescribed burning is commonly used throughout the region to maintain various pine ecosystems including, longleaf pine ecosystems. We selected 21 sites (two privately owned, eight state-owned, and 11 owned by the J.W. Jones Ecological Research Center [JERC]) based on the presence or absence of a long-term history of prescribed burning, with burned sites having had at least 10 years of regular prescribed burns and unburned sites having no history of prescribed burning for over 10 years (Table 1). Study sites (not including our control sites) ranged in size from 7.2–78.9 ha. To ensure that the same locations within the study sites, including our control sites, were being sampled on a month-to-month basis, specific plots were established which ranged from 0.35–3.22 ha (note that flagging effort was standardized by time). No permits were required for this work. Permission to work on public lands was given by the Georgia Department of Natural Resources. Permission to work on private lands was given by the respective land owners and future permissions should be addressed to Mike Conner.

**Table 1:** Locations and burn data for the 21 plots sampled during this study.

Location	GPS Coordinates	County†	Burn Treatment	Year Burned (during study)
JERC, BU 1*	N 31° 18.967, W 84° 26.495	Baker	BB	2010
JERC, BU 5	N 31° 18.422, W 84° 27.177	Baker	BB	2011
JERC, BU 7 East	N 31° 17.272, W 84° 27.877	Baker	BB	2011
JERC, BU 7 West	N 31° 17.770, W 84° 28.396	Baker	BB	2011
JERC, BU 64	N 31° 25.042, W 84° 48.285	Baker	BB	2010
JERC, BU 136	N 31° 11.627, W 84° 27.978	Baker	BB	2010
JERC, BU 137	N 31° 11.594, W 84° 27.976	Baker	BB	2011
JERC, BU 176	N 31° 14.484, W 84° 22.162	Baker	BB	2010
Chickasawhatchee WMA	N 31° 29.055, W 84° 26.744	Calhoun	BUB	2010
Hannahatchee WMA - plot 1	N 32° 8.850, W 84° 45.241	Stewart	BUB	2011
Hannahatchee WMA - plot 2	N 32° 06.559, W 84° 44.600	Stewart	BUB	2011
River Creek WMA	N 30° 51.023, W 84° 04.347	Thomas	BUB	2011
Silver Lake WMA	N 30° 47.270, W 84° 45.390	Decatur	BUB	2010
Flint River WMA	N 32° 08.431, W 84° 00.358	Dooly	UBB	n/a
JERC, BU 107	N 31° 16.367, W 84° 29.090	Baker	UBB	n/a
JERC, BU 140	N 31° 10.219, W 84° 28.017	Baker	UBB	n/a
JERC, BU 15	N 31° 29.066, W 84° 52.242	Baker	UBB	n/a
Lake Seminole WMA - Little Horseshoe Bend Tract	N 30° 50.649, W 84° 39.186	Decatur	UBB	n/a
Montezuma Bluff Natural Area	N 32° 20.235, W 84° 01.743	Macon	UBUB	n/a
Private Property 1	N 30° 49.578, W 84° 36.951	Decatur	UBUB	n/a
Private Property 2	N 30° 41.277, W 84° 49.023	Gladsden, Florida	UBUB	n/a

Prescribed burns took place every 2–4 years during the dormant season on pre-determined schedules.

JERC =  Joseph W. Jones Ecological Research Center; BU =  burn unit; WMA =  wildlife management area; n/a =  not applicable; BB =  burned, surrounded by burned area; BUB =  burned, surrounded by unburned area; UBB =  unburned, surrounded by burned area; UBUB =  unburned, surrounded by unburned area.

*JERC plots were distributed throughout this 12,000 ha area.

† Unless noted, all counties are in Georgia.

To account for differences in land cover and management both within and immediately surrounding the site, each site was further categorized as being 1) burned surrounded by burned (BB), 2) burned surrounded by unburned (BUB), 3) unburned surrounded by burned (UBB) and 4) unburned surrounded by unburned (UBUB) (i.e., a control). When classifying prescribed fire status around a sampled site, surrounding land was defined based on burn status of land immediately surrounding the site. Because of the rarity of BUB sites, we classified these sites based upon the majority of the land immediately surrounding the plot being unburned. Selection of BUB and UBB was made possible by using or working adjacent to sites that were either part of a long-term fire exclusion site or using sites that were not burned due to logistical difficulties associated with smoke dispersion (e.g., stands bordered highways creating risk of automobile accident), or selecting sites associated with forest types that are not typically burned (e.g., hardwood forest). Common use of prescribed fire on the study landscape facilitated selection of BB sites. Lack of prescribed fire use within large tracts of land facilitated selection of UBUB sites (with tracts ranging from 165–385 ha).

### Tick Collections

Each plot was sampled monthly for ticks for 24 months (January 2010-December 2011) using 1 m×1 m flags made of flannel cloth. To standardize conditions, flagging was conducted only when the temperature was above 7.2°C and after 10AM when vegetation was dry (no dew or moisture from precipitation). Additionally, flagging was not conducted during inclement weather (rain, snow, or excessive wind). Each plot was flagged for a minimum of one hour per plot per month. If more than 5 ticks were collected during a one hour effort, sampling would be continued for up to 30 minutes or until the entire plot had been covered. Flags were checked every 10 minutes and all nymphs and adults were collected and preserved in 70% ethanol. All larvae were removed en masse with masking tape with each clutch being kept separate. Timers were paused during flag checks and removal of ticks.

### Tick Identifications

All adult and nymphal *Amblyomma* spp., adult *Dermacentor variabilis*, and adult *Ixodes* spp. were identified morphologically utilizing a microscope and published keys [33], [34]. Identification of larvae, *Ixodes* spp. nymphs, and a subset of adult *Ixodes* spp. was conducted through polymerase chain reaction (PCR) and sequence analysis. DNA was extracted from ticks using a Qiagen DNeasy blood and tissue kit (Germantown, MD) utilizing the manufacturer's protocol. For *Amblyomma* larvae collected in 2010, a multiplex real-time PCR was used to differentiate between *A. maculatum* and *A. americanum* as described in Zemtsova et al [35]. For *Amblyomma* larvae collected in 2011 and all *Ixodes*, a PCR targeting the 16S rRNA gene was conducted using primers 16S-1 (5′-CCGGTCTGAACTCAGATCAAGT) and 16S+2 (5′-TTGGGCAAGAAGACCCTATGAA) as described in Black and Piesman [36]. DNA extraction and PCR reactions were performed in hoods dedicated strictly for their respective tasks and waters were included as negative controls during both DNA extraction and PCR in order to detect contamination. All amplicons from the 16S protocol were purified using a Qiagen gel extraction kit (Germantown, MD) and then bi-directionally sequenced at the Georgia Genomics Facility (Athens, GA).

### Host Monitoring

Host occurrence, specifically mesomammals and deer, was determined through quarterly passive trail camera (Cuddeback Capture, Green Bay, WI) surveys. No permits or Institutional Animal Care and Use Committee approval is required for passive trail camera surveys and no endangered or protected species were involved. For each survey, one camera was placed at each plot on an unbaited, secondary dirt road or trail bordering or within 100 m of the plot. Each plot was surveyed for 76 hours with all plots being surveyed within an 11-day period (with the exception of the winter 2011 survey in which all plots were surveyed within 30 days due to logistical constraints). The species, date, and time of all animals captured were recorded.

### Vegetation Surveys

To account for potential differences in vegetation among the plots, vegetation surveys were performed at all plots. To determine tree density, a singular point-centered quarter survey utilizing the equivalent of 20 points per hectare was performed with a quarter being considered empty if there were no trees within 10 m from the center point. These points were marked for understory vegetation surveys.

Understory vegetation and canopy closure were measured using quarterly surveys. To perform the survey, a 1 m×1 m frame was placed with the previously marked point falling at the center of the frame and the sides of the frame running directly north to south and east to west. Percent cover and composition of understory vegetation and ground litter was estimated [37]. Litter depth was measured at two random points within the frame. Canopy cover was measured from the center point using a spherical densiometer [38].

### Microclimate and Weather

At least three microclimatic measurements (humidity, temperature, and median wind speed) were taken at each plot immediately after each monthly flagging using a Kestrel 3000 Weather Meter (Birmingham, MI). Measurements were taken at ground level and 1 m above ground. If a particular plot had areas with and without ticks, measurements were taken where ticks were collected and two additional measurements were taken at randomly assigned points where ticks were not detected. If no ticks were collected at a plot, measurements were taken at randomly selected points.

Weather data including maximum temperature and precipitation the current sampling day, maximum and minimum temperatures the previous day, and precipitation accumulation the previous 3 days were collected for all plots, each sample date from the JERC Weather Station (all JERC locations) and the National Weather Service (all other plots).

### Statistical Analyses

A generalized estimating equation (GEE) negative binomial regression model [39] was used to evaluate the impacts of long-term prescribed burning, host abundance, vegetation composition, microclimate, and weather on tick abundance. This modeling approach is well-suited for repeated measures, accounts for clustering by plot, and is appropriate for use when there are large numbers of zeros in the data. Models were created for 1) total tick counts, 2) *A. americanum* counts, and 3) *I. scapularis* counts. We chose to include all lifestages captured by flagging because this method of collection acts as a proxy for questing behavior and ultimately the ability to locate a human host which has direct implications for pathogen transmission. When applicable, each larval cluster was counted as a single tick because 1) this would reduce skew in the data, and 2) larvae do not transmit pathogens to humans (except for *Rickettsia* spp.). However, we chose to include nymphs in our analyses because they are viewed as the primary lifestage responsible for transmitting pathogens to humans [40], [41]. No other tick species were sufficiently abundant for further modeling effort.

Because host and vegetation surveys were only conducted quarterly, all data were evaluated on a quarterly basis (spring, summer, fall, and winter). Thus, for tick counts, microclimate, and weather data, we used data that were collected closest to the date of the vegetation surveys for each quarter. The relationship between continuous predictors and the outcome was graphically assessed by categorizing the predictors into quartiles and plotting the estimated coefficients for the resulting indicator variables against the category midpoints. Predictors having a non-linear relationship with the outcome were categorized for the analysis.

Multivariable model selection proceeded from a maximum model that contained all variables having an association (*P*<0.2) with tick counts in the univariate analysis. Non-significant predictors were removed from the maximum model using a manual stepwise procedure until only those having *P*<0.05 remained. Upon reaching a preliminary main-effects model, all previously excluded variables were added back to the final model one at a time to re-assess their significance, and all possible two-way interactions with burning were evaluated. Year and season were retained regardless of their significance because of their theoretical importance as predictors of tick abundance. Season was dichotomized into warm (spring and summer) and cool (fall and winter) periods because the individual seasons within these categories were homogeneous with respect to tick counts.

## Results

**Figure 1:**
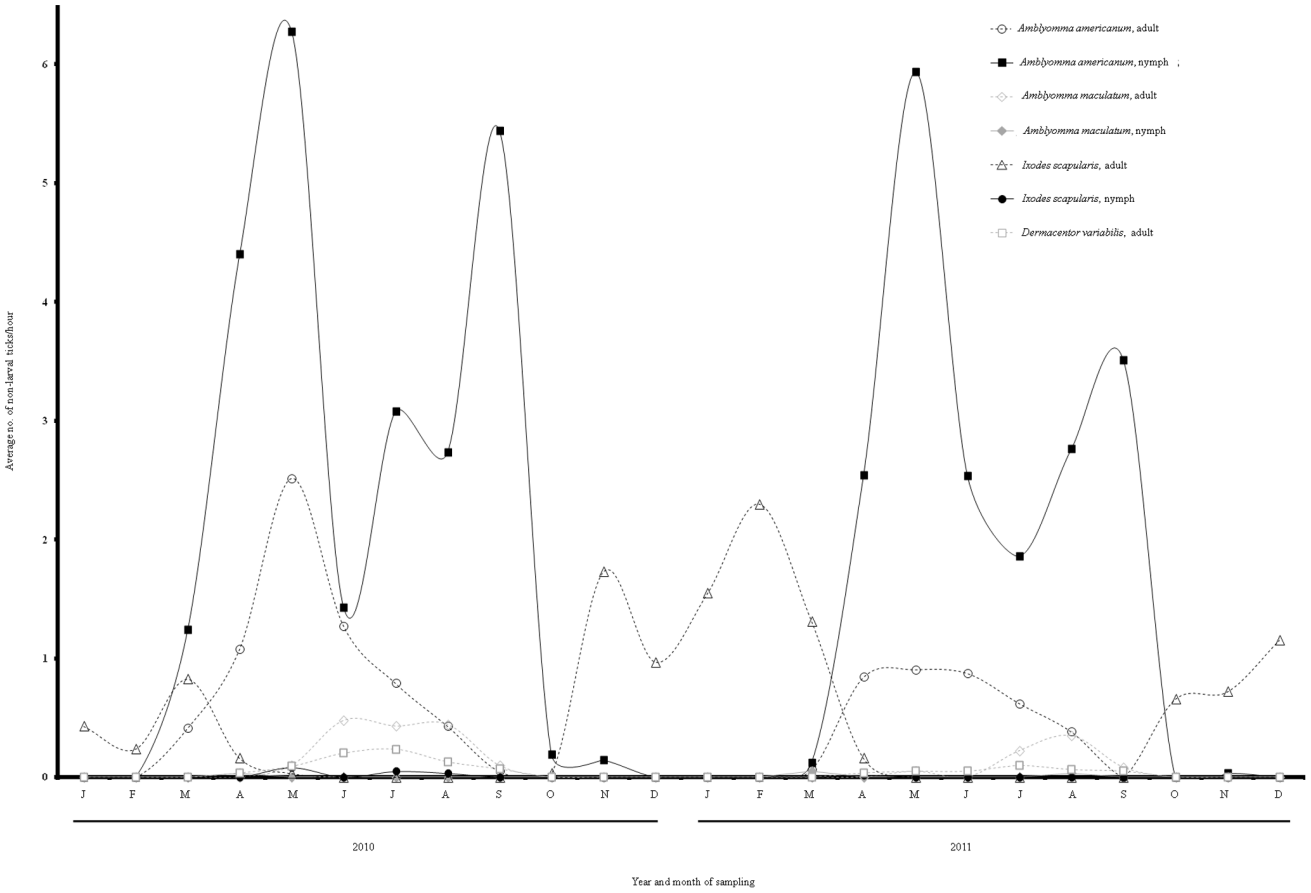
Seasonality of each tick species and life stage across all study plots for 2010 and 2011.

In total, 47,184 ticks were collected with *A. americanum* being the most common tick detected followed by *I. scapularis, A. maculatum, D. variabilis*, and *I. brunneus* (Table 2). To account for varying effort among plots and collection dates, we standardized by evaluating ticks collected/hr. Seasonality trends were determined using all 21 plots for both 2010 and 2011 (Figure 1). In 2010, *A. americanum* activity peaked in May for adults, May and October for nymphs, and July and September for larvae. In 2011, periods of peak *A. americanum* activity were similar with adults peaking in April through June, nymphs in May and September, and larvae in July and October. Adult *A. maculatum* activity peaked June through August 2010 and in August 2011 (Figure 1). Single *A. maculatum* nymphs were collected in April 2010 and in March 2011 and a single *A. maculatum* larval clutch was collected in September 2011. *I. scapularis* adults peaked in November and March of 2010 and in February 2011. *D. variabilis* adult activity peaked in July 2010 and was not caught in enough abundance in 2011 to determine seasonality.

**Table 2:** Number of ticks collected from different burn regimes during 2010 and 2011.

	Treatment	adult		nymph		larvae (clutches)	
		2010	2011	2010	2011	2010	2011
*A. americanum*	BB	0	1	0	0	0	0
	BUB	4	7	2	21	1,362 (1)	291 (2)
	UBB	5	4	24	4	2,354 (2)	1,177 (5)
	UBUB	272	170	1,171	981	26,432 (147)	12,433 (72)*
*A. maculatum*	BB	30	6	1	0	0	0
	BUB	25	6	0	1	0	0
	UBB	3	2	0	1	0	0
	UBUB	0	3	0	0	0	unknown*
*I. scapularis*	BB	2	3	0	0	0	0
	BUB	8	18	0	0	0	0
	UBB	40	94	1	0	0	0
	UBUB	47	128	5	1	5 (1)	0
*D. variabilis*	BB	0	0	0	0	0	0
	BUB	9	2	0	0	0	0
	UBB	7	2	0	0	0	0
	UBUB	13	5	0	0	0	0

BB = Burned, surrounded by burned, BUB =  Burned, surrounded by unburned, UBB =  Unburned, surrounded by unburned, UBUB =  Unburned, surrounded by unburned.

*1 group of 40 larvae (not included in the numbers in this table) thought to be a single clutch was found to contain both *A. americanum* and *A. maculatum* upon PCR testing.

**Figure 2:**
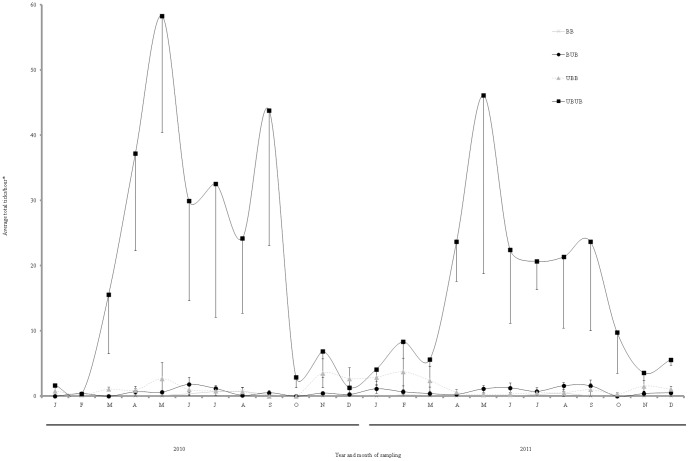
Effects of long-term prescribed burning on tick abundance at all study plots. *One clutch of larvae was counted as one tick.

Within burn treatments, 43 ticks were collected in BB plots (one *A. americanum*, 37 *A. maculatum,* and five *I. scapularis*), 1,756 in BUB plots (1,687 *A. americanum,* 32 *A. maculatum*, 26 *I. scapularis,* and 11 *D. variabilis*), 3,719 in UBB plots (3,568 *A. americanum*, six *A. maculatum*, 135 *I. scapularis,* one *I. brunneus*, and nine *D, variabilis*), and 41,706 in UBUB plots (a minimum of 41,460 *A. americanum*, a minimum of four *A. maculatum*, 186 *I. scapularis*, and 18 *D. variabilis*) (Table 2). Importantly, species-specific numbers associated with *A. americanum* and *A. maculatum* in UBUB plots cannot be determined because a larvae cluster was captured that was assumed to consist of a single species; later PCR testing revealed that it contained both species. Our modeling efforts suggested that total tick counts were affected by long-term burning, season, litter cover, and trees density (Table 3). There was a significant interaction between burning and season. For plots in which burning occurred, tick counts did not change significantly by season [RR = 1.1 (95% CI: 0.57, 2.12); P = 0.774], confirming the trend that ticks were significantly reduced in burned plots (Figure 2). However, at plots in which there was no burning, tick counts were 10 times greater in the warm season than in the cold [Relative Risk (RR)  = 10.7 (95% CI: 4.20, 27.18); P<0.001]. Having higher than 95% litter cover was positively associated with an approximately two-fold increase in tick counts and density of trees (>183 per ha) was associated with an approximately six-fold increase in tick counts.

**Table 3:** Generalized estimating equation negative binomial regression model for the prediction of total tick counts at all 21 plots in 2010 and 2011.

Variable	Coefficient (SE)	RR (95% CI)	P
Any Burning (vs. No Burning*)	−1.5 (0.43)	ND	0.001
2011 (vs. 2010*	0.29 (0.24)	1.3 (0.84, 2.2)	0.22
Hot (Spring/Summer) (vs. Cool [Fall/Winter]*)	2.4 (0.48)	ND	<0.001
>95% Litter Cover (vs. <95%*)	0.81 (0.33)	2.2 (1.2, 4.3)	0.014
>183 Trees per Ha(vs. <183*)	2.3 (0.66)	6.4 (2.6, 35)	0.001
Any Burning X Season	−2.3 (0.59)	ND	<0.001
Constant	−2.0 (0.84)	NA	0.016
ln(Hours flagged)	1		

SE =  Standard error. RR =  Relative rate. ND =  Not determined; RR is not given because it depends on the interacting variable. NA =  Not applicable.

*Indicates the reference category.

**Figure 3:**
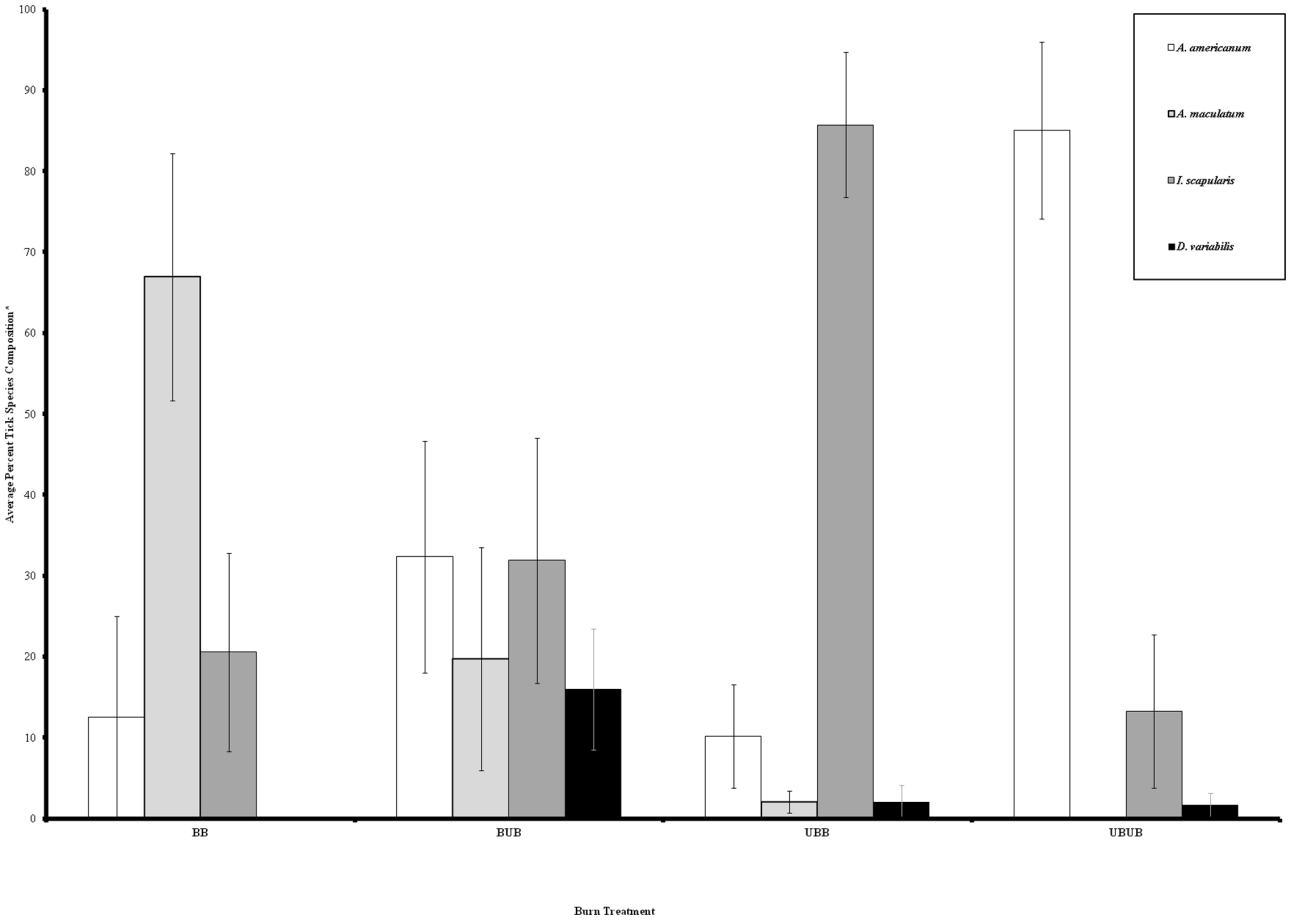
Ticks species composition at all study plots by burn treatment for 2010–2011. *One clutch of larvae was counted as one tick.

Interestingly, dominant tick species differed by burn treatment, with *A. americanum* being the most prevalent tick in UBUB plots, *A. maculatum* being most common in BB plots, *I. scapularis* dominating UBB plots, and a more even distribution of the different tick species at BUB plots (Figure 3). To further investigate impacts of the measured variables on individual tick species, we constructed models specifically for *A. americanum* (Table 4) and *I. scapularis* (Table 5). *A. americanum* counts were best predicted by burning, season, bobcat presence/absence, and wind at ground level on day of tick counts. A significant interaction occurred between burning and season, with *A. americanum* not being significantly affected by season in burned plots [RR = 2.4 (95% CI: 0.3, 19.0); P = 0.409]; in other words, no seasonality was observed because there were so few ticks present in burned plots. However, at plots in which there was no burning, *A. americanum* counts were over 30 times greater in the warm season than in the cold [RR = 33 (95% CI: 14, 81); P<0.001], indicating what would be an expected seasonality pattern for *A. americanum*. Together, these findings suggest that prescribed burning reduced *A. americanum* abundance. The presence of bobcats (*Lynx rufus*) was positively associated with *A. americanum* counts, while an increase in wind by 1 kilometer per hour resulted in a 69% decrease in *A. americanum* detectability. Finally, *I. scapularis* counts were best predicted by burning, season, year, tree density, and precipitation. Long-term prescribed burning reduced *I. scapularis* counts by 78%. Furthermore, *I. scapularis* counts were reduced by approximately 85% in the warm season relative to the cold season and counts were 3.7 times greater in 2011 than in 2010. Lastly, *I. scapularis* counts were 18 times greater in plots with high tree density (>183 trees per ha) relative to plots with low tree density and were twice as likely to be collected if precipitation occurred during the 3 days prior to sampling.

**Table 4:** Generalized estimating equation negative binomial regression model for the prediction of *A. americanum* counts at all study 21 plots.

Variable	Coefficient (SE)	RR (95% CI)	P
Any Burning (vs. No Burning*)	−2.5 (1.1)	ND	0.026
2011 (vs. 2010*)	0.07 (0.34)	1.1 (0.55, 2.1)	0.837
Hot (Spring/Summer) (vs. Cold [Fall/Winter]*)	3.5 (0.45)	ND	<0.001
Bobcats Present (vs. Absent)	1.2 (0.31)	3.2 (1.7, 5.8)	<0.001
Wind at Ground (mph)	−1.2 (0.34)	0.31 (0.15, 0.59)	<0.001
Any Burning X Season	−2.6 (1.1)	ND	0.019
Constant	1.3 (0.84)	NA	0.717
ln(Hours flagged)	1	(Effort)	

SE =  Standard error. RR =  Relative rate. ND =  Not determined; RR is not given because it depends on the interacting variable. NA =  Not applicable.

*Indicates the reference category.

**Table 5:** Generalized estimating equation negative binomial regression model for the prediction of *I. scapularis* counts at all 21 plots in 2010 and 2011.

Variable	Coefficient (SE)	RR (95% CI)	P
Any Burning (vs. No Burning*)	−1.2 (0.40)	0.29 (0.13, 0.63)	0.002
2011 (vs. 2010*)	1.3 (0.42)	3.8 (1.6, 8.7)	0.002
Hot (Spring/Summer) (vs. Cold [Fall/Winter]*)	−1.9 (0.47)	0.15 (0.06, 0.37)	<0.001
>183 Trees per Ha (vs.<183*)	2.9 (0.98)	18 (2.6, 122)	0.003
>0 cm Precipitation Previous 3 Days (vs. 0 cm*)	0.74 (1.1)	2.1 (1.2, 3.8)	0.014
Constant	−3.6 (1.1)	NA	0.001
ln(Hours flagged)	1	(Effort)	

SE =  Standard error. RR =  Relative rate. NA =  Not applicable.

*Indicates the reference category.

## Discussion

To our knowledge, this is one of the most comprehensive studies examining the effects of long-term operational prescribed burning while also incorporating other variables that are known to affect tick abundance. Furthermore, this is the first long-term study of tick phenology in southwestern Georgia, and provides valuable insight into seasonality and species composition within common habitats of the region.

Gaining a better understanding of tick species composition and seasonality in this rarely studied region provides important information for public health education and research. Our findings regarding seasonality of ticks were generally in agreement with studies done in other regions of Georgia and northern Florida [24], [27]. One exception was that we detected a bi-modal peak for *A. americanum* nymphal activity similar to work conducted by Cilek and Olson [27] in northern Florida, whereas only a single peak was noted by Davidson et al. [24] in the Piedmont region of Georgia (i.e., farther north). This may indicate that nymphal *A. americanum* populations south of the fall line in Georgia have bi-modal peaks, whereas those north of the fall line have a single peak. Also, to our knowledge, this marks only the second longitudinal study on *A. maculatum* seasonality [42], providing important information on this tick which has gained interest in recent years due to expanding populations, transmission of numerous pathogens, and having been identified as a vector of the causative agent of *Rickettsia parkeri* rickettsiosis [43].

Past studies suggested that climate (e.g. temperature and rainfall) affects tick abundance and distribution [24], [44] and we suspect that the differences observed in tick phenology from 2010 to 2011 were a result of climate differences. In 2011 there were significantly more *I. scapularis.* Furthermore, the bi-modal peak of *I. scapularis* occurred later in 2011 as compared to 2010. These differences may have been due to an exceptionally dry summer followed by a wet fall and mild winter (*I. scapularis* adult peak activity) in 2011 as compared to 2010.

Long-term prescribed burning significantly reduced tick counts. Although some previous studies have found either increases in tick populations or a recovery of tick populations following a burn [19]–[28], our results suggest that prior conclusions may be the result of failure to account for other variables and failure to simulate real-world management scenarios in which large-scale burns are regularly applied. Because operational burns typically occur on larger areas, re-colonization by ticks in these larger areas may take longer than observed in studies using smaller plots. Furthermore, repeated burn events occurring over time may deplete source populations of ticks.

Interestingly, we observed variation in the tick community composition relative to burn treatment which, to our knowledge, has never been reported. The most striking difference was related to greater abundances of *A. maculatum* in BB plots whereas *A. americanum* was most abundant in UBUB plots. In general, *A. americanum* are more abundant in hardwood forests [45], and some have suggested that *A. americanum* outcompetes other tick species, such as *D. variabilis* [46]. Thus, the greater abundances of *A. americanum* in UBUB plots which were hardwood dominated would arguably be expected. The greater question deals with the absence of *A. americanum* and dominance of *A. maculatum* in BB plots. Burning at BB plots directly altered the physical vegetation structure and maintained dominance of pines in the overstory. Thus, BB plots were generally open-canopied pine forests with a diverse understory and sparse midstory. These vegetation conditions result in a hotter, dryer environment than UBUB plots which are closed-canopy hardwood forests with dense mid-stories and sparse, litter-covered understories. We suspect that this plays a key role in both the long-term reduction of ticks and shift in tick species composition observed at these plots. Our models support this as vegetation structure (tree density and litter cover specifically) and microclimatic variables which would affect tick moisture retention were most important in predicting tick counts. Willis et al. [30] had similar findings in that *A. americanum* was found to be associated with litter cover and negatively associated with pine sapling density. Also in support of this theory, Gleim et al. [47] reported that *A. americanum* had significantly lower survival in experimental enclosures in burned habitats vs. unburned habitats compared with *A. maculatum*. Collectively, these data suggest that different physiologic and/or behavioral traits of different tick species increase survival success in certain environments [47], [48].

Although host occurrence (specifically mesomammals and deer) was considered in all models of tick counts, only bobcat presence was an important predictor of *A. americanum* counts, suggesting that those host populations monitored did not generally impact tick counts. It is important to note, however, that our methodology (trail camera surveys) did not account for small mammal and bird populations which are important hosts for both *A. americanum* and *A. maculatum* larvae and nymphs [49]–[51]. However, previous studies have found that small mammals and ground nesting birds tend to be found in greater abundance in burned areas where there is dense ground cover as compared to unburned areas in which ground cover is sparse to non-existent [52], [53]. These data would therefore refute the assertion that the high tick abundances observed in UBUB were due to larger populations of small mammals and birds in these areas.

The failure to identify a relationship between host occurrence and tick counts questions acceptance of the dogma that host abundance drives tick abundance [54]–[56]. However, manipulative and observational studies have been inconclusive regarding a correlation between host and tick abundance [57], [58]. Although bobcat presence was an informative predictor of *A. americanum* counts, we suggest that it is more likely bobcats were associated with other factors impacting *A. americanum* counts rather than actually acting as a causal mechanism affecting *A. americanum* counts. We suspect that while host abundance may play a minor role in tick dynamics, other factors such as burning and microclimate play larger roles in driving tick dynamics in the systems we studied.

In conclusion, operational prescribed burning in pine and mixed pine ecosystems significantly reduces tick populations which supports several previous studies [20], [26]. A number of previous studies showed that although burning causes tick mortality, they recolonized the area within a few years [24], [25], [30]. We suggest that the repeated burnings associated with operational prescribed fires and the resulting vegetation and microclimate are ultimately responsible for the long-term reductions in tick populations observed in our study. These findings illustrate another benefit of prescribed fire and have important implications for public health as it is a time efficient, cost effective method for reducing tick populations and likely reduces the risk of human tick-borne diseases.

Future studies should include pathogen testing to gain a more complete understanding of tick-borne disease risk and dynamics in areas that utilize prescribed burns. Furthermore, studies examining the efficacy of prescribed burning in different management scenarios as well as determining the length of time required to provide overall reductions in tick populations within previously unburned areas is warranted.
